# Reply: “Comorbidity between epilepsy and headache/migraine: the other side of the same coin!”

**DOI:** 10.1007/s10194-011-0378-5

**Published:** 2011-08-23

**Authors:** Irene Toldo, Pier Antonio Battistella

**Affiliations:** Department of Pediatrics, Juvenile Headache Center, University of Padua, Via Giustiniani, 3, Padova, 35128 Italy

Dear Sir,

We thank Dr. Striano and co-workers for their interest in our two recently published papers on comorbidity between headaches, in particular migraine, and epilepsy [1–3]. Dr. Striano underlies the importance of neuronal hyperexcitability and increased susceptibility to cortical spreading depression, sustained by both genetic and non-genetic factors, as the main pathophysiological mechanism of this association, accordingly to other authors [2–4].

Dr. Striano correctly points out that large well-phenotyped samples and massive genotyping will give a chance to discover the genetic factors involved in the comorbidity between headache/migraine and epilepsy [1]. The two main aspects that complicate the analysis of the molecular mechanisms underlying the comorbidity between headache/migraine and epilepsy are: (1) that while some epileptic syndromes are monogenic, apart from the exception of hemiplegic migraine, common forms of migraine are multifactorial and polygenic diseases; (2) the neuronal depolarization that gives rise to a seizure and the cortical spreading depression that causes migraine share some pathophysiological mechanisms.

We believe that some clinical markers (such as the presence of EEG photosensitivity, osmophobia, family history of epilepsy and headache, migraine aura) could help to select more homogeneous populations of patients for genotype analysis, therefore it is important to collect cases, prospectively, with extensive phenotypic characterization. For this purpose well-designed multicenter studies are desirable to achieve a high sample size.

Another important point raised by Dr. Striano is the need to revise the international classification of both epilepsy and headache disorders and to introduce widely accepted and more precise diagnostic criteria of migralepsy, hemicrania epileptica, post-ictal headache and “ictal epileptic headache” [1, 5, 6].

Further studies are necessary to clarify the pathopshysiological mechanisms of the association between headache/migraine and epilepsy.
